# Ceramide Production Mediates Aldosterone-Induced Human Umbilical Vein Endothelial Cell (HUVEC) Damages

**DOI:** 10.1371/journal.pone.0146944

**Published:** 2016-01-20

**Authors:** Yumei Zhang, Yu Pan, Zhixiang Bian, Peihua Chen, Shijian Zhu, Huiyi Gu, Liping Guo, Chun Hu

**Affiliations:** Division of Nephrology, Shanghai Ninth People’s Hospital, Shanghai JiaoTong University School of Medicine, Shanghai, 201999, China; Ottawa Hospital Research Institute, CANADA

## Abstract

Here, we studied the underlying mechanism of aldosterone (Aldo)-induced vascular endothelial cell damages by focusing on ceramide. We confirmed that Aldo (at nmol/L) inhibited human umbilical vein endothelial cells (HUVEC) survival, and induced considerable cell apoptosis. We propose that ceramide (mainly C18) production might be responsible for Aldo-mediated damages in HUVECs. Sphingosine-1-phosphate (S1P), an anti-ceramide lipid, attenuated Aldo-induced ceramide production and following HUVEC damages. On the other hand, the glucosylceramide synthase (GCS) inhibitor PDMP or the ceramide (C6) potentiated Aldo-induced HUVEC apoptosis. Eplerenone, a mineralocorticoid receptor (MR) antagonist, almost completely blocked Aldo-induced C18 ceramide production and HUVEC damages. Molecularly, ceramide synthase 1 (CerS-1) is required for C18 ceramide production by Aldo. Knockdown of CerS-1 by targeted-shRNA inhibited Aldo-induced C18 ceramide production, and protected HUVECs from Aldo. Reversely, CerS-1 overexpression facilitated Aldo-induced C18 ceramide production, and potentiated HUVEC damages. Together, these results suggest that C18 ceramide production mediates Aldo-mediated HUVEC damages. MR and CerS-1 could be the two signaling molecule regulating C18 ceramide production by Aldo.

## Introduction

Recent studies have implied an important role of aldosterone (Aldo) in the pathogenesis of multiple vascular diseases [1,2]. Studies have demonstrated that plasma Aldo level is significantly elevated in the vascular disease patients [1,2]. Exogenous infusion of Aldo could induce direct deleterious effect on vascular tissues in animal models [3,4,5,6,7,8]. Vascular endothelial cell apoptosis is recognized as the fundamental step in the progression of vascular sclerosis [9]. Yet, the underlying molecular mechanisms are not fully studied. To this regard, Aldo was added to cultured vascular epithelial cells, and associated signaling changes were analyzed [10,11]. In the current study, we studied the possible role of ceramide in the process.

Existing evidences have established ceramide as an important player in apoptosis induction [12,13,14]. A number of cytotoxic agents were shown to induce ceramide production, mediating following cell death [12,13,14]. Increased ceramide production in multiple human cell lines could lead to growth inhibition, cell apoptosis, differentiation and senescence [15,16]. Anti-cancer chemotherapeutic agents, including taxol, doxorubicin as well as several natural compounds were shown to induce cellular ceramide production, mediating subsequent cell apoptosis [15,16].

In this study, we hypothesized that Aldo-induced vascular endothelial cell apoptosis may be accompanied with increased ceramide production, which might contribute significantly to vascular cell damages. To test this hypothesis, we examined the cellular ceramide level in Aldo-treated human umbilical vein endothelial cells (HUVECs). Pharmacological and genetic strategies were applied to alter cellular ceramide level, Aldo-induced HUVEC cytotoxicity in these conditions was tested.

## Material and Methods

### 2.1. Chemicals, reagents and antibodies

Aldosterone (Aldo), L-threo-1-phenyl-2-decanoylamino-3-morpholino-1-propanol (PDMP) sphingosine-1-phosphate (S1P) and Eplerenone were obtained from Sigma-Aldrich Chemicals (Sigma, St. Louis, MO). The cell-permeable short chain Ceramide (C6) was obtained from Avanti Polar Lipids, Inc. (Alabaster, AL). The pan-caspase inhibitor z-VAD-fmk, the caspase-3 specific inhibitors z-DVED-fmk and AC-DEVD-CHO were purchased from Calbiochem (Shanghai, China). All the antibodies utilized in this study were obtained from Abcam (Danvers, MA).

### 2.2. HUVEC culture

Human umbilical vein endothelial cells (HUVECs) were isolated from human umbilical cord veins by collagenase I (0.25%, Sigma) digestion. The harvested cells were grown in medium 199 (Gibco, Shanghai, China) containing 15% heat-inactivated fetal calf serum (FCS, Gibco), endothelial cell growth supplement (ECGS, 30 μg/mL, Sigma), epidermal growth factor (EGF 10 ng/mL, Sigma), 100 U/mL penicillin, and 100 μg/mL streptomycin. After 3–5 passages, HUVECs were collected for experimental use. All research involving human samples have been approved by the Shanghai Ninth People’s Hospital, Shanghai JiaoTong University School of Medicine Institutional Review Board (IRB) members: Wang Li, Jing Li, Gang Wu and Xiao-jing Li. The approval number is NO2013025. All clinical investigation has been conducted according to the principles expressed in the Declaration of Helsinki. Informed written consents have been obtained from each participants.

### 2.3. MTT cell survival assay

HUVEC viability was measured by the 3-[4,5-dimethylthylthiazol-2-yl]-2,5 diphenyltetrazolium bromide (MTT, Sigma) assay. Briefly, HUVECs were seeded onto 96-well plate at a density of 3 x 10 ^3^ cells/well. After applied treatments, twenty μL/well of MTT tetrazolium solution (5 mg/mL) was added, and incubated in a CO_2_ incubator for additional 3 hours. Finally, the medium was aspirated, and 150 mL/well of DMSO (Sigma) was added to dissolve formazan crystals. The absorbance of each well was obtained using a plate reader at the wavelength of 490 nm. OD value was utilized as an indicator of cell viability.

### 2.4. Lactate dehydrogenase (LDH) assay

LDH content released to the conditional medium indicates the level of cell death. After applied treatment, HUVEC medium LDH was assayed by a LDH detection kit from Roche Applied Science (Shanghai, China). LDH release % = LDH released in conditional medium/(LDH released in conditional medium + LDH in cell lysates) x 100%. HUVECs were lysed by 1% Triton X-100.

### 2.5. Fragmented DNA detection by ELISA

Nucleosomal DNA fragmentation is one biological marker of cell apoptosis [17]. Fragmented DNA was assessed by measuring DNA-associated with nucleosomal histones through a specific two-site ELISA with an anti-histone primary antibody, and a secondary anti-DNA antibody, according to the manufacturer's instructions (Roche Applied Science, Shanghai, China). ELISA OD at 450 nm was recorded as a quantitative measurement of HUVEC apoptosis.

### 2.6. Annexin V FACS assay of cell apoptosis

Apoptosis was detected by an Annexin-V-FITC apoptosis detection kit (BD Pharmingen, San Diego, CA). Briefly, following applied treatment, HUVECs were harvested and washed twice with cold PBS, and then incubated for 15 min with Annexin-V-FITC and propidium iodide (PI). Both early (Annexin V^+^/PI^−^) and late (Annexin V^+^/PI^+^) apoptotic cells were gated by the fluorescence activated cell sorter (FACS) machine (BD Pharmingen). The percentage of Annexin V stained HUVECs was utilized as another quantitative measurement of cell apoptosis.

### 2.7. Caspase-3 activity assay

After applied treatment, cytosolic proteins of approximately 1 × 10^6^ HUVECs were extracted. Thirty μg of cytosolic extracts per sample were added to caspase assay buffer (312.5 mm HEPES, pH 7.5, 31.25% sucrose, 0.3125% CHAPS) with benzyloxycarbonyl-DEVD-7-amido-4-(trifluoromethyl)coumarin as the substrate (Calbiochem). After 2 hours of incubation at 37°C, the release of 7-amido-4-(trifluoromethyl)coumarin (AFC) was quantified, using a Fluoroskan system (Thermo-Labsystems, Helsinki, Finland) set to an excitation value of 355 nm and emission value of 525 nm as described [18].

### 2.8. Western blot

After treatment, the cells were harvested with trypsinization, centrifuged and lysed in lysis buffer (Biyuntian, Wuxi, China). Total protein was quantified by the Bio-Rad assay kit, mixed with 5 times sample buffer and boiled at 95°C for 5 min. Equal amount of proteins (30 μg/sample) were separated by electrophoresis in SDS-PAGE, transferred to the PVDF membrane, and were detected with the specific antibody. The immuno-reactive proteins after incubation with appropriately labeled secondary antibody were detected with an enhanced chemiluminescence (ECL) detection kit (Amersham, Buckinghamshire, UK). Band intensity was quantified by ImageJ software (NIH) after normalization to the corresponding loading control.

### 2.9. Enzymatic measurement of total cellular ceramide

The total cellular ceramide level was analyzed with the help from Dr. Ming Xu’ lab at Tongji University (Shanghai, China) utilized the 1,2-diacylglycerol (DAG) kinase method as described [19,20], and was valued as fmol by nmol of phospholipid. Its level in the treatment group was expressed as the fold change of the untreated control group. Each measurement was performed triplicate.

### 2.10. Liquid chromatography-mass spectrum (LC-MS) detection of individual cellular ceramide

Individual ceramide production was detected by LC-MS method as previously described [21,22]. Briefly, after treatment, cells were resuspended in PBS, and the lipids were extracted with ethyl acetate/isopropanol/water (60/30/10, v/v) [21,22]. Extracted lipids were then dried under N_2_, which was solubilized in 56.7% methanol, 33.3% ethanol, 10% water, and derivatized with *ortho*-phthaldialdehyde (Sigma) [21]. Thereafter, the lipids were separated on a C_18_ column [21,22] and analyzed by a serial arrangement of Hypersil C8/150×3.2 mm column followed by a mass spectrum (MS) detector (Thermo Finnigan TSQ 7000 triple quadrupole mass spectrometer) operating in a multiple reaction monitoring positive ionization mode. Non-natural individual ceramides (C18/C20/C22/C24) (Avanti) were utilized as the internal standards. Results were normalized to total phospholipid contents and expressed as pmol ceramide/nmol phosphate.

### 2.11. Ceramide synthase 1 (CerS-1) shRNA knockdown

To knockdown ceramide synthase 1 (CerS-1), CerS-1-shRNA (h) lentiviral particles (sc-62543-V, Santa Cruz Biotech, Santa Cruz, Ca) (10 μL/mL medium) were added directly to HUVECs, the infection took 48 hours. Afterwards, the CerS-1 expression was verified by Western blot. Control cells were infected with same amount of scramble shRNA lentiviral particles (sc-108080-V, Santa Cruz) (10 μL/mL medium, 48 hours).

### 2.12. Ceramide synthase 1 (CerS-1) over-expression

The full-length human CerS-1 cDNA was synthesized by Genechem (Shanghai, China). The cDNA was inserted into pSuper-puro plasmid (Youbio, Beijing, China). The CerS-1 plasmid or the empty vector (pSuper-puro) was then transfected into HEK-293 cells with plasmids encoding viral packaging proteins VSVG and Hit-60 (Promega) [23] using Lipofectamine 2000 (Invitrogen). The virus-containing supernatants were collected and filtered, and were then added to HUVECs for 48 hours. CerS-1 over-expression in infected cells was confirmed by Western blots.

### 2.13. Statistics

The results were expressed as the mean ± standard deviation (SD). Statistical significance (p<0.05) was evaluated by one-way ANOVA followed by Bonferroni post hoc test (SPSS 15.0, Chicago, IL).

## Results

### 3.1. Aldosterone exerts cytotoxic effects to cultured HUVECs

**Fig 1:**
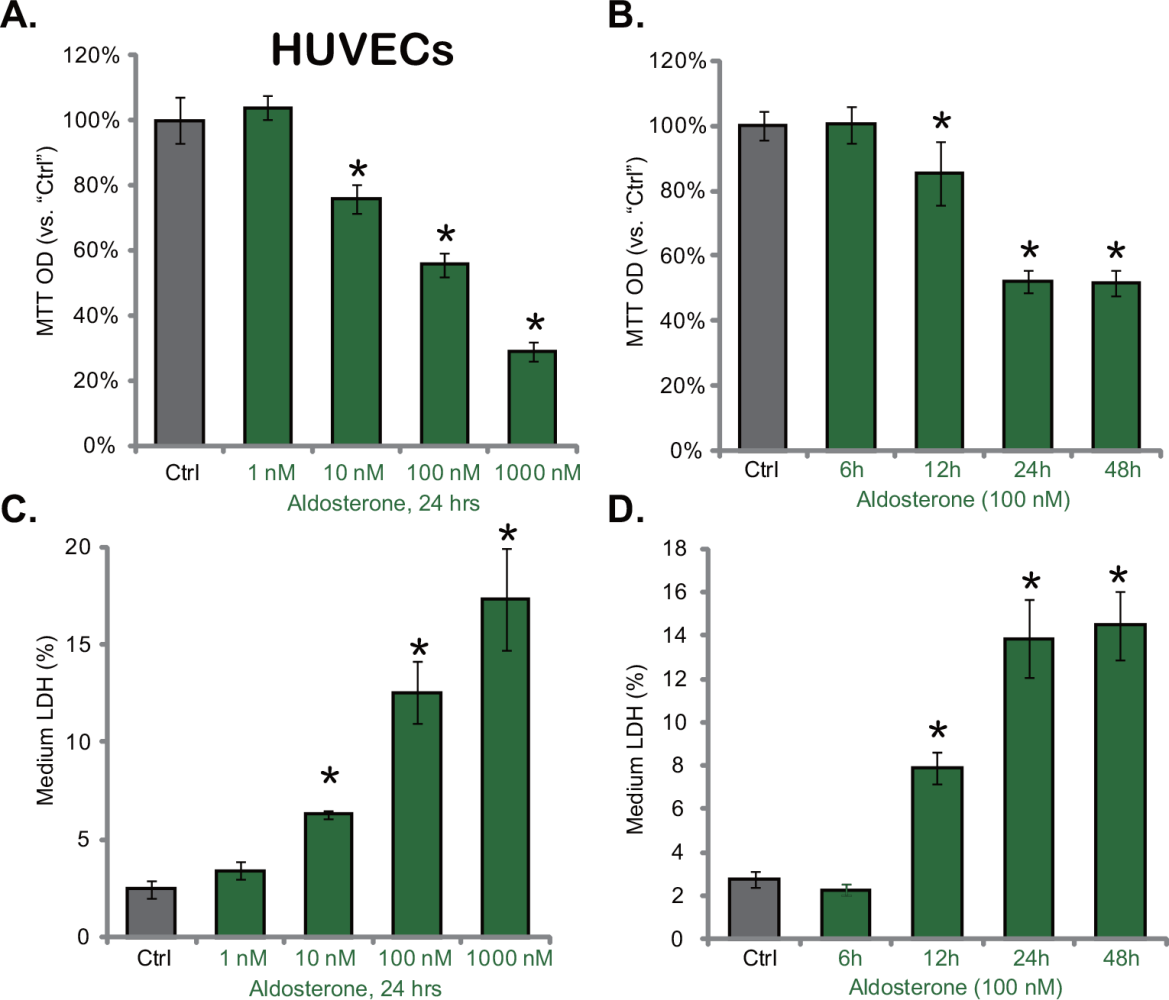
Aldosterone exerts cytotoxic actions to cultured HUVECs. HUVECs were either left untreated (“Ctrl”), or treated with applied concentrations of aldosterone (1–1000 nM) for indicated time point, cell survival was tested by MTT assay (A and B), and cell death was tested by LDH release assay (C and D). Data were expressed as the mean ± SD. For each assay, n = 5. Experiments in this figure were repeated four times, and similar results were obtained. * p < 0.05 vs. “Ctrl” group.

We first examined the potential effect of Aldo on cultured vascular cells. Primary HUVECs were treated with indicated concentrations of Aldo, cells were future cultured. MTT cell viability results in Fig 1A demonstrated that Aldo dose-dependently inhibited HUVEC survival. HUVEC viability OD was 75.8 ±4.6%, 55.5 ± 3.5% and 29.0 ± 2.9% of untreated control after 10, 100 and 1000 nM of Aldo treatment, respectively (Fig 1A). Further, Aldo at 100 nM showed a time-dependent manner in inhibiting HUVEC survival (Fig 1B). A significant viability reduction was observed 24 and 48 hours after Aldo treatment (Fig 1B). At the meantime, the level of medium LDH of Aldo-treated HUVECs was significantly increased, suggesting cell death (Fig 1C and 1D). These results together demonstrate that Aldo is cytotoxic when added directly to cultured HUVECs.

### 3.2. Aldosterone induces caspase-3-dependent apoptotic death in HUVECs

**Fig 2:**
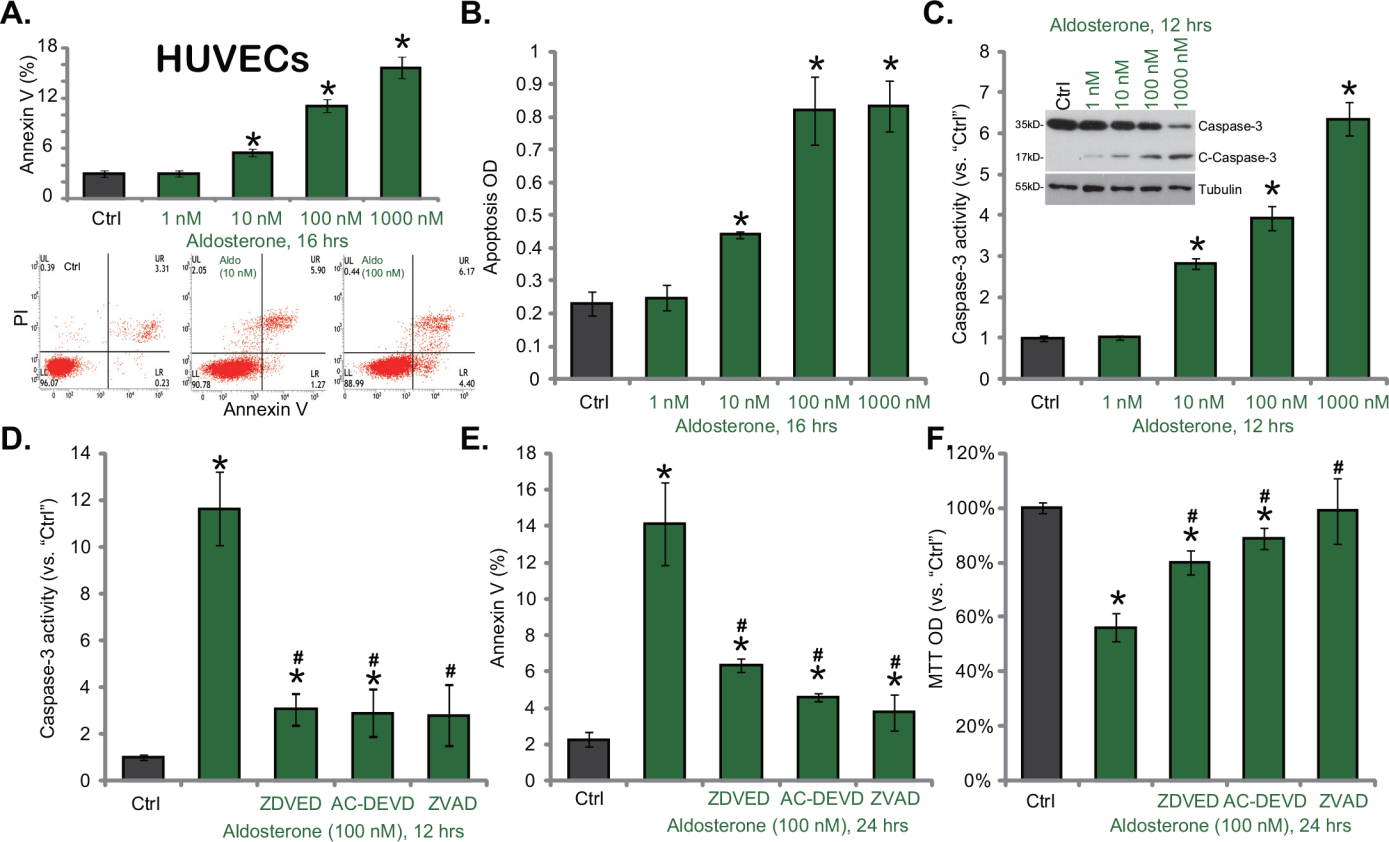
Aldosterone induces caspase-3-dependent apoptotic death in HUVECs. HUVECs were treated with applied concentrations of aldosterone (1–1000 nM) for indicate time, cell apoptosis was evidenced by Annexin V FACS assay (A, representative FACS images were shown in lower panel), histone DNA ELISA assay (B), caspase-3 activity assay (C, lower panel) and Western blot assaying of cleavead-caspase-3 (“C-Caspase-3”) (C, upper panel). HUVECs, pre-treated with the caspase-3 inhibitor z-DVED-fmk (“ZDVED”, 25 μM)/AC-DEVD-CHO (“AC-DEVD”, 25 μM), or the pan caspase inhibitor z-VAD-fmk (“ZVAD”, 25 μM) for 1 hour, were stimulated with aldosterone (100 nM), caspase-3 activity and cell apoptosis were analyzed by the caspase-3 activity assay (D) and Annexin V FACS assay (E), respectively; Cell survival was tested by the MTT assay (F). Data were expressed as the mean ± SD. For each assay, n = 5. Experiments in this figure were repeated three times, and similar results were obtained. * p < 0.05 vs. “Ctrl” group. ^#^ p < 0.05 vs. aldosterone (100 nM) only group (D-F).

Next, we studied the potential effect of Aldo on HUVEC apoptosis. Three independent apoptosis assays were performed. Results demonstrated clearly that Aldo dose-dependently induced apoptosis in HUVECs (Fig 2A–2C). The percentage of Annexin V positive cells was significantly increased following Aldo (10–1000 nM, 24 hours) treatment (Fig 2A). Fig 2A lower panel demonstrated representative Annexin V FACS images of HUVECs before (“Ctrl”) and after Aldo (10/100 nM, 24 hours) treatment. The apoptosis histone-DNA ELISA OD (Fig 2B) and the caspase-3 activity (Fig 2C, lower panel) were also increased with Aldo (100–1000 nM) treatment in HUVECs. In addition, the level of cleaved-caspase-3 was significantly increased following Aldo treatment in HUVECs, while regular caspase-3 level was decreased (Fig 2C, upper panel). These results indicate that Aldo induced significant apoptosis activation in HUVECs.

To investigate the potential role of apoptosis activation in Aldo-induced HUVEC cytotoxicity, various caspase inhibitors were utilized: including the specific caspase-3 inhibitors (z-DVED-fmk and AC-DEVD-CHO), and the pan caspase inhibitor (z-VAD-fmk). Results showed that these inhibitors almost completely blocked Aldo-induced caspase-3 activation and following HUVEC apoptosis (Fig 2D and 2E). As a result, Aldo-induced HUVEC cytotoxicity, evidenced by viability OD reduction, was remarkably attenuated with the co-treatment of the caspase inhibitors (Fig 2F). These caspase inhibitors alone had no effect on HUVEC survival or apoptosis (Data not shown). Thus, Aldo induces caspase-3-dependent apoptosis in cultured HUVECs.

### 3.3. Aldosterone mainly induces C18 ceramide production in HUVECs

**Fig 3:**
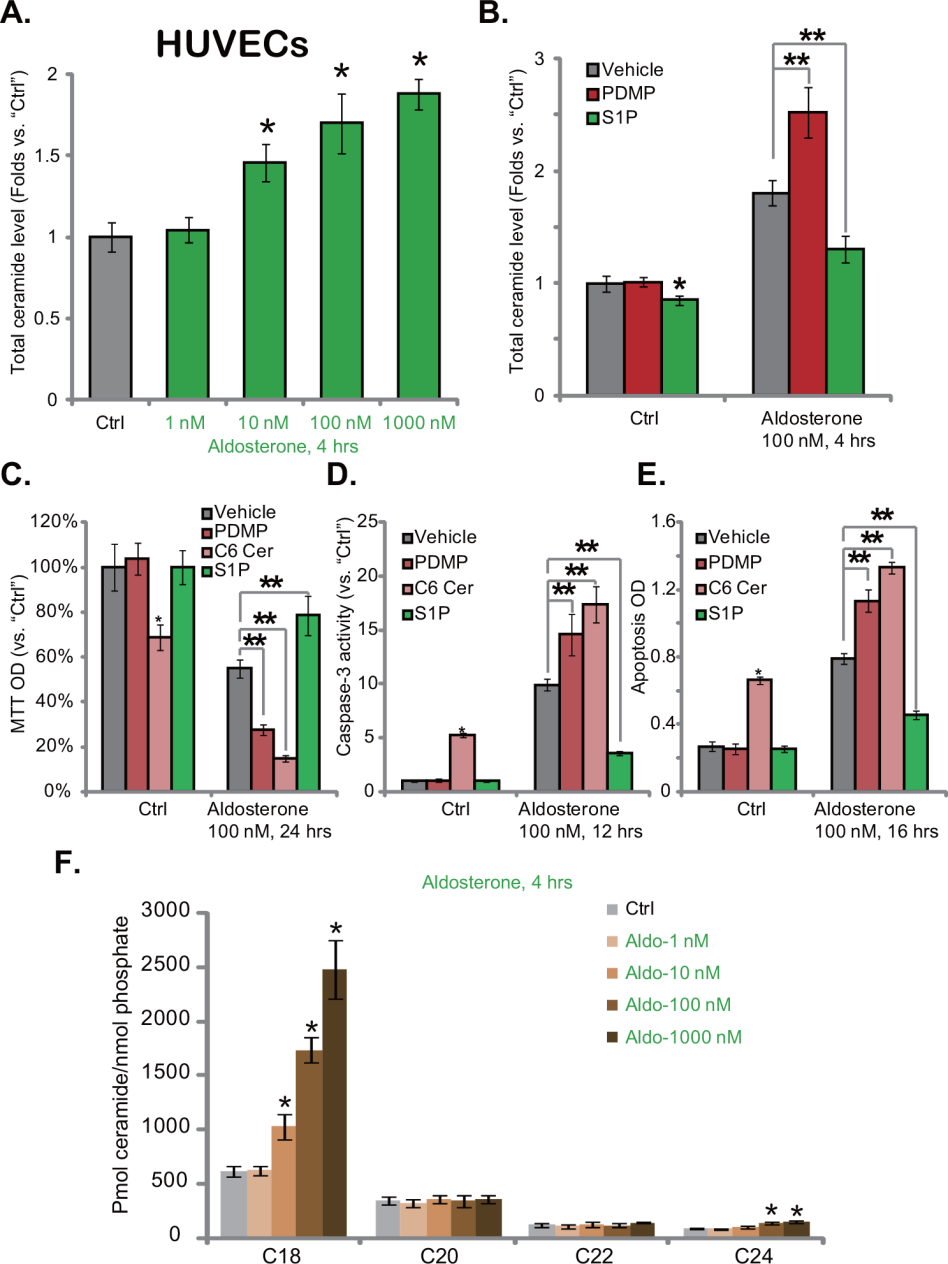
Aldosterone mainly induces C18 ceramide production in HUVECs. HUVECs were treated with applied concentrations of aldosterone (1–1000 nM) for 4 hours, total cellular ceramide level was analyzed by the DAG kinase assay, and was normalized to the untreated control (“Ctrl”) group (A), individual ceramide level was detected by LS-MS assay as described (F). HUVECs, pretreated with PDMP (10 μM), S1P (10 μM) or C6 ceramide (25 μM) for 1 hour, were stimulated with aldosterone (100 nM), cellular ceramide was analyzed (B); Cell survival was tested by MTT assay (C), and cell apoptosis was tested by the caspase-3 activity assay (D) or the Histone DNA ELISA assay (E). For each assay, n = 3. Experiments in this figure were repeated three times, and similar results were obtained. * p < 0.05 vs. “Ctrl” group. ** p < 0.05 (C-E).

We next aimed to understand the potential role of ceramide in Aldo-induced vascular cell damages. At first, diacylglycerol (DAG) kinase assay [20] was performed to determine total cellular ceramide. Results in Fig 3A demonstrated that Aldo induced ceramide production in HUVECs (Fig 3A), the level of total cellular ceramide was significantly increased following Aldo (10–1000 nM) treatment (Fig 3A). To study the role of ceramide in Aldo-induced apoptosis, pharmacological strategy was applied. As shown in Fig 3B, Aldo (100 nM)-induced ceramide production in HUVECs was inhibited by sphingosine-1-phosphate (S1P, a sphingosine counteracting ceramide’s effect [24]), but was potentiated by PDMP, which is a glucosylceramide synthase (GCS) inhibitor [25] (Fig 3B). Remarkably, Aldo (100 nM)-induced cytotoxicity (Fig 3C) and apoptosis (tested by caspase-3 activity and apoptosis ELISA assay, Fig 3D and 3E) were significantly inhibited by S1P, but were exacerbated by PDMP (Fig 3C–3E). Further, the actions by Aldo in HUVECs were mimicked by the exogenously-added cell-permeable ceramide (C6) (Fig 3C–3E). In addition, ceramide (C6) also enhanced Aldo-mediated HUVEC cytotoxicity (Fig 3C–3E). Together, these results indicate that ceramide production is important for Aldo-mediated HUVEC damages.

Next, we wanted to know which individual ceramide was induced by Aldo in HUVECs. The liquid chromatography-mass spectrum (LC-MS) method was applied (See method). As shown in Fig 3F, C18 was the major individual ceramide in HUVECs. It level was significantly increased following Aldo treatment (10–1000 nM) (Fig 3F). Level of other individual ceramide, including C20, C22 and C24, was dramatically lower than that of C18 (Fig 3F). Among these individual ceramide, only C24 ceramide level was slightly increased following Aldo (100/1000 nM) treatment in HUVECs (Fig 3F). Other individual ceramide (C12, C14 etc) level was even lower (Data not shown). There results indicate that Aldo mainly induces C81 ceramide production in HUVECs.

### 3.4. Eplerenone blocks aldosterone-induced ceramide production and following HUVEC cytotoxicity

**Fig 4:**
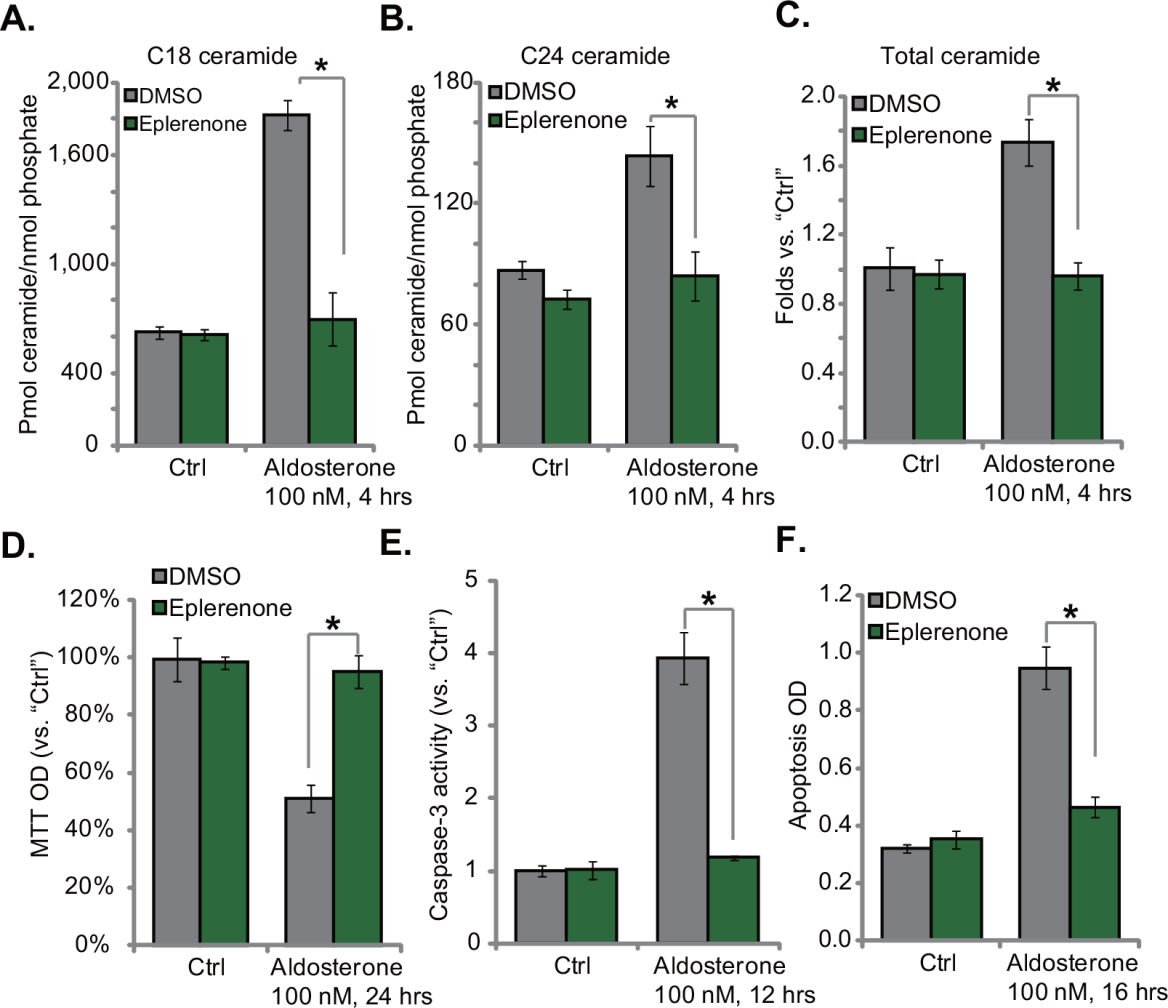
Eplerenone blocks aldosterone-induced ceramide production and following HUVEC cytotoxicity. HUVECs, pretreated with Eplerenone (10 μM) for 1 hour, were stimulated with aldosterone (100 nM), C18 ceramide (A, LS-MS assay), C24 ceramide (B, LS-MS assay), and total ceramide (C, DAG kinase assay) were analyzed after 4 hours; Cell viability was tested by MTT assay (D), and cell apoptosis was tested by the caspase-3 activity assay (E) and Histone DNA ELISA assay (F). For each assay, n = 3. Experiments in this figure were repeated three times, and similar results were obtained. * p < 0.05.

Next, we studied the molecular mechanisms underlying ceramide production by Aldo. Eplerenone, an Aldo mineralocorticoid receptor (MR) antagonist [26], was applied. Results demonstrated that Eplerenone almost completely blocked Aldo-induced C18, C24 and total ceramide production in HUVECs (Fig 4A–4C), indicating a critical role of MR in mediating ceramide production by Aldo. As a result, Aldo-induced HUVEC death (Fig 4D, viability reduction) and apoptosis (Fig 4E and 4F, tested by caspase-3 activity assay and histone-DNA ELISA assay) were significantly inhibited by the MR antagonist. These results indicate that functional MR is required for Aldo-induced ceramide production and HUVEC damages.

### 3.5. Ceramide synthase 1 mediates aldosterone-induced C18 ceramide production and following HUVEC damages

**Fig 5:**
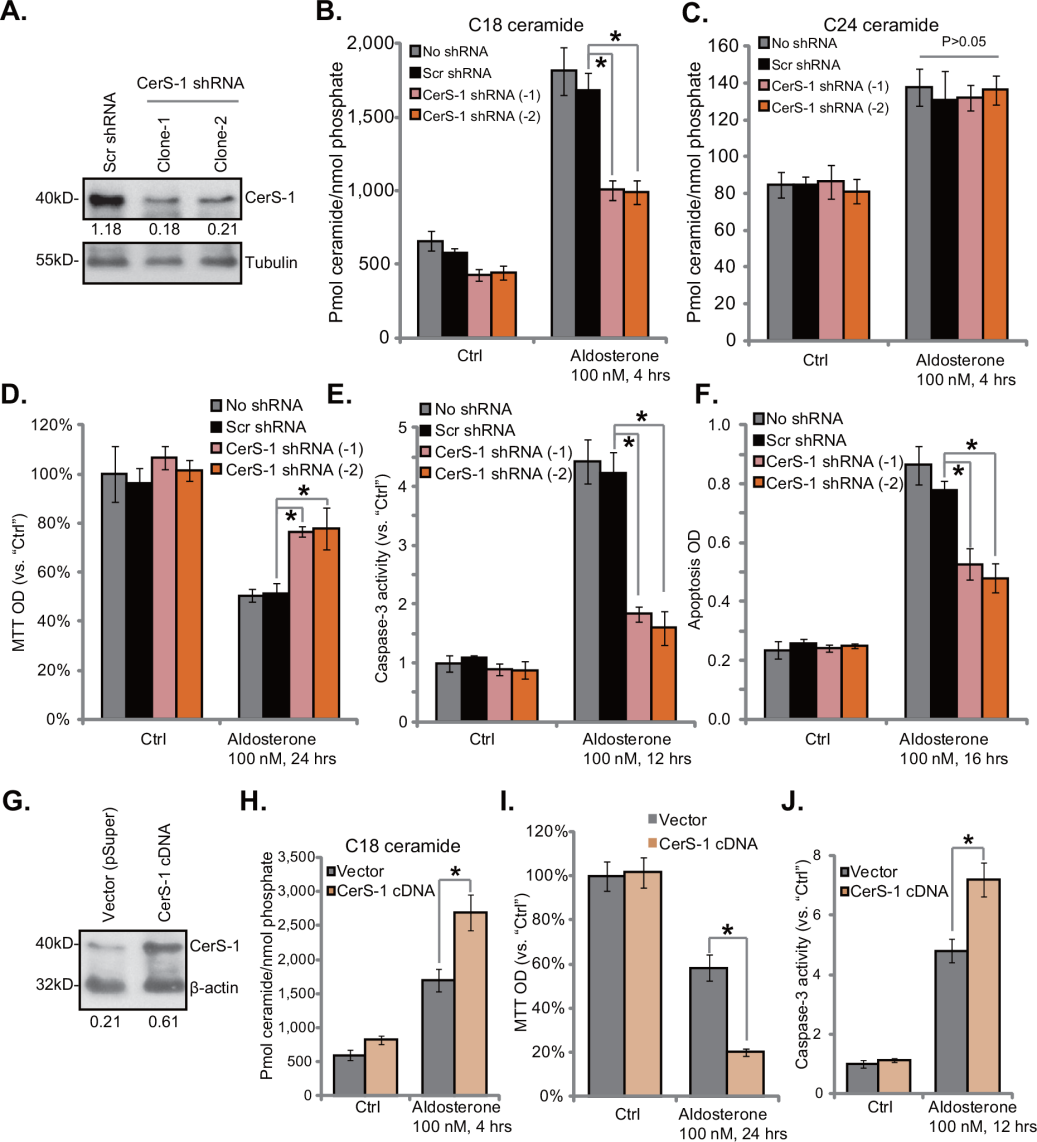
Ceramide synthase 1 mediates aldosterone-induced C18 ceramide production and following HUVEC damages. Expression of CerS-1 and tubulin (the equal loading) in HUVECs expressing scramble control shRNA or CerS-1 shRNA (two colonies) was shown (A), CerS-1 expression (vs. tubulin) was quantified (A). Above cells were treated with or without aldosterone (100 nM), C18 and C24 ceramide production (B and C), cell survival (D) and cell apoptosis (Caspase-3 activity, E and Histone DNA EILSA OD, F) were tested. Expression of CerS-1 and β-actin (the equal loading) in HUVECs expressing CerS-1-cDNA or the empty vector (p-Super-puro) was shown, CerS-1 expression (vs. β-actin) was quantified (G); Aldo (100 nM)-induced C18 ceramide production (H, 4 hours), cell viability reduction (I, 24 hours), and caspase-3 activity (J, 12 hours) in above cells were tested. For each assay, n = 3. Experiments in this figure were repeated three times, and similar results were obtained. * p < 0.05.

The above results demonstrated that Aldo mainly induced C18 ceramide production in HUVECs. Ceramide synthase 1 (CerS-1) is a key enzyme responsible for C18 ceramide synthesis [27]. We thus tested whether CerS-1 was also involved in Aldo-mediated C18 ceramide production. The shRNA method was applied. Western blot results in Fig 5A showed that expression of CerS-1 was remarkably downregulated by targeted CerS-1 shRNA. Two CerS-1 shRNA-expressing HUVEC clones (-1/-2) were selected (Fig 5A). Notably, Aldo (100 nM)-induced C18 ceramide production was remarkably inhibited by CerS-1 shRNA (Fig 5B). On the other hand, Aldo-mediated C24 ceramide production was not affected by CerS-1 knockdown (Fig 5C). Importantly, Aldo-exerted HUVEC viability reduction (Fig 5D) and apoptosis (Fig 5E and 5F) were significantly attenuated with CerS-1 knockdown. These results indicate that CerS-1 and it-mediated C18 ceramide production is critical in regulating Aldo’s actions in HUVECs.

To further confirm the role of CerS-1 in Aldo-mediated cytotoxicity, over-expression strategy was utilized. We successfully constructed a CerS-1-cDNA plasmid (see method). Results in Fig 5G demonstrated CerS-1 over-expression in the HUVECs with the plasmid. CerS-1 over-expression facilitated Aldo-induced C18 ceramide production (Fig 5H). As a result, Aldo-exerted HUVEC death (viability reduction, Fig 5I) and apoptosis (caspase-3 activation, Fig 5J) were potentiated with CerS-1 over-expression (Fig 5G). Together, these results again suggest that CerS-1 is important for Aldo-induced C18 ceramide production and HUVEC cytotoxicity.

## Discussions

Ceramide is a well-known apoptosis mediator in response to various cytotoxic stimuli [28,29]. In the current study, we showed that ceramide production is also required for Aldo-mediated deleterious effects in cultured HUVECs. S1P attenuated Aldo-induced ceramide production, thus inhibiting following HUVEC damages. Eplerenone, a mineralocorticoid receptor (MR) antagonist, almost completely blocked Aldo-induced (C18/C24) ceramide production and subsequent HUVEC death/apoptosis. Importantly, exogenously-added cell-permeable ceramide (C6) was shown to mimic Aldo’s actions, and induced significant HUVEC damages. Molecularly, CerS-1 could be the enzyme for C18 ceramide production by Aldo. CerS-1 knockdown attenuated Aldo-induced C18 ceramide production, and protected HUVECs from Aldo. CerS-1 over-expression, on the other hand, sensitized Aldo-induced C18 ceramide production and HUVEC apoptosis. Together, these results suggest that ceramide (mainly C18) production is critical for Aldo-mediated HUVEC damages.

Aldo is a key regulator of blood pressure and electrolytic balance. Recent evidences, however, have demonstrated that increased Aldo could exert a number of deleterious effects to the cardiovascular system, including myocardial necrosis and fibrosis, vascular stiffening, reduced fibrinolysis as well as endothelial dysfunction and injuries, catecholamine release, and cardiac arrhythmias [2,3,30,31,32,33]. Thus, excessive Aldo contributes to the development and progression of a number of cardiovascular diseases, including hypertension, congestive heart failure, chronic kidney disease, coronary artery disease, and stroke [2,3,30,34]. Aldo has displayed a number of direct actions to epithelial cells [32,35,36]. In the current study, we showed that Aldo activated MR-CerS-1 pathway to induce (C18) ceramide production, mediating HUVEC damages.

Studies have shown that Aldo activated two pathways to exert its action. One is the classic MR pathway, and the other is the novel MR-independent pathway [30]. Here, we showed that Aldo-induced (C18) ceramide production and HUVEC damages were almost completely blocked by the MR antagonist Eplerenone, suggesting the pivotal role of classic MR pathway in the process. Yet, besides ceramide production, activation of MR by Aldo in epithelial cells could exert a number of other actions, including activation of several downstream signalings (i.e. endoplasmic reticulum stress and ERK-MAPK) [37] and production of reactive oxygen species (ROS) [36]. Further studies will be needed to explore the detailed signaling mechanisms of CerS-1 activation/ceramide production by Aldo-MR. In particularly, if there is a link between CerS-1 activation and other known actions of Aldo. More importantly, it will also be critical to know if ceramide production could affect other endpoints of Aldo in epithelial cells, including epithelial-to-mesenchymal transition (EMT) [36], production of vascular endothelial growth factor (VEGF) [35] and adhesion molecule [30,38], as well as vascular nitric oxide (NO) bioavailability inhibition [39,40].

Cells are capable of removing excess ceramide through various metabolic clearance pathways [41,42]. As a matter of fact, agents that inhibit metabolically clearance of ceramide could lead to a pro-apoptotic outcome [43,44,45,46]. In the current study, we showed that co-administration of PDMP, the glucosylceramide synthase (GCS) inhibitor [46,47], facilitated Aldo-induced ceramide accumulation, therefore enhancing HUVEC apoptosis and cytotoxicity. Other studies using similar strategies showed that PDMP sensitized cytotoxicity by several chemo-agents (Taxol [48],Vincristine [48] and curcumin [46]) in different cancer cells. These results further confirm the involvement of ceramide in Aldo-induced actions in HUVECs.

## Conclusions

In conclusion, our results suggest that ceramide (mainly C18) production mediates Aldo-induced damages to cultured HUVECs. Functional MR and CerS-1 could be the signaling molecule regulating C18 ceramide production by Aldo. Amelioration of Aldo-induced (C18) ceramide production could be a novel strategy for the treatment of Aldo-associated vascular disease.
